# 0094. Monocyte tace activity profile during sepsis and systemic inflammatory response syndrome

**DOI:** 10.1186/2197-425X-2-S1-P6

**Published:** 2014-09-26

**Authors:** DJ O'Callaghan, KP O'Dea, M Takata, AC Gordon

**Affiliations:** Imperial College London, London, UK; Imperial College Healthcare National Health Service Trust, London, UK

## Introduction

To date, immune-modulatory treatments in sepsis have proved unsuccessful. Better understanding of inflammatory processes might help tailor such therapies more effectively and improve their efficacy. It is now recognised that some patients may require immune stimulating therapies.

Monocytes display altered behaviour in response to systemic inflammation; this has been called “deactivation” and is characterised by reduced HLA-DR expression and attenuated tumour necrosis factor-α (TNF) release in response to lipopolysaccharide (LPS) stimulus^1^. TNF converting enzyme (TACE), activated via p38-mitogen activated protein kinase (MAPK) mediated pathway^2^, is essential for the shedding of TNF and TNF receptors (TNFRs)-1&2. Altered TACE behaviour may be mechanistically important in deactivation.

## Objectives

To determine whether monocyte TACE activity is affected by sepsis and is reflective of illness severity.

## Methods

Sixteen patients with sepsis, 15 healthy volunteers and 8 patients with non-infectious systemic inflammatory response syndrome (NI-SIRS) were recruited and data collected at baseline (D0), day 2 (D2) and day 4 (D4). Peripheral blood mononuclear cells were separated by density gradient centrifugation with HLA-DR, TACE and TNFR expression (basal and LPS-induced) determined by flow cytometry. LPS-induced TNF release was measured using ELISA. Monocytes were isolated using magnetic bead selection and TACE catalytic activity (basal and LPS-induced) was measured using fluorescence resonance energy transfer assay^3^.

**Figure 1:**
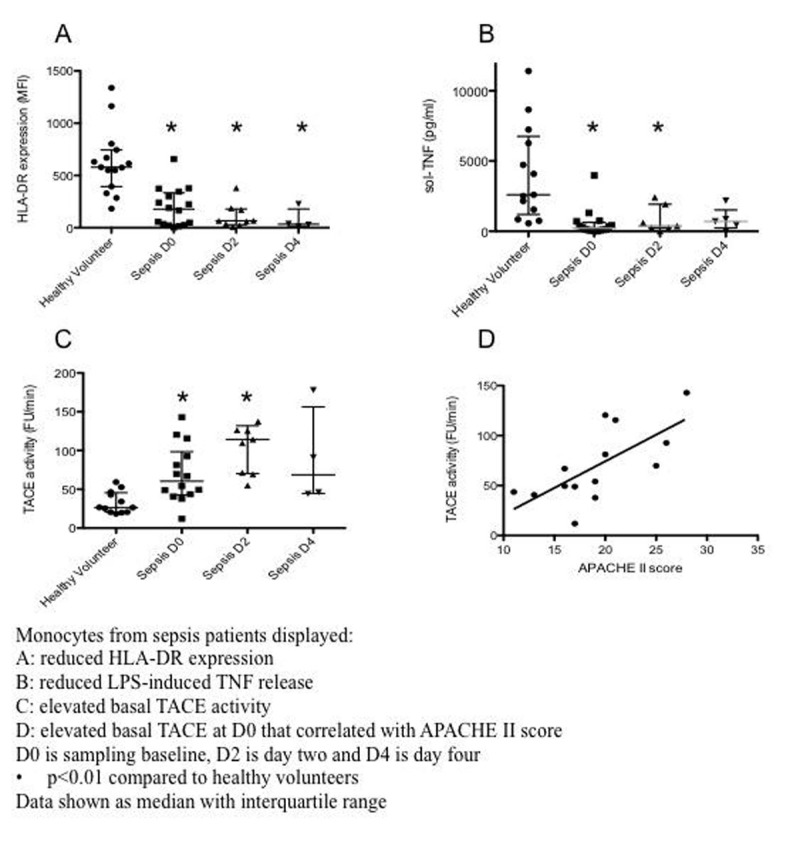
Figure 1

## Results

HLA-DR expression and LPS-induced monocyte TNF release were attenuated in sepsis and NI-SIRS (p< 0.01), consistent with deactivation. Sepsis, but not NI-SIRS, produced elevated basal monocyte TACE activity (p< 0.01) while LPS-induced increases in TACE activity were attenuated. Elevated D0 basal TACE activity positively correlated with APACHE II score in sepsis (p< 0.01), but not NI-SIRS.

TACE expression was unaltered across all groups, but LPS-induced TNFR shedding appeared reduced by sepsis. Sepsis patients displayed an attenuated p38-MAPK response to LPS.

## Conclusions

Our data indicate that sepsis induces specific changes in monocyte TACE catalytic activity profiles that may reflect illness severity, whilst such changes were not seen in NI-SIRS. These changes in TACE activity attenuated LPS-induced release of TNFR and TNF from monocytes, and appear to be mediated through altered upstream p38-MAPK signalling.
